# Identifying Knowledge Gaps through the Systematic Review of Temperature-Driven Variability in the Competence of *Aedes aegypti* and *Ae. albopictus* for Chikungunya Virus

**DOI:** 10.3390/pathogens12111368

**Published:** 2023-11-18

**Authors:** Rebecca C. Christofferson, Erik A. Turner, Víctor Hugo Peña-García

**Affiliations:** 1 School of Veterinary Medicine, Louisiana State University, Baton Rouge, LA 70803, USA; eturn35@lsu.edu; 2 Department of Biology, Stanford University, Stanford, CA 94305, USA; vhpena@stanford.edu

**Keywords:** chikungunya, extrinsic incubation period, EIP, extrinsic incubation temperature, EIT, transmission, *Aedes aegypti*, *Aedes albopictus*, temperature

## Abstract

Temperature is a well-known effector of several transmission factors of mosquito-borne viruses, including within mosquito dynamics. These dynamics are often characterized by vector competence and the extrinsic incubation period (EIP). Vector competence is the intrinsic ability of a mosquito population to become infected with and transmit a virus, while EIP is the time it takes for the virus to reach the salivary glands and be expectorated following an infectious bloodmeal. Temperatures outside the optimal range act on life traits, decreasing transmission potential, while increasing temperature within the optimal range correlates to increasing vector competence and a decreased EIP. These relatively well-studied effects of other *Aedes* borne viruses (dengue and Zika) are used to make predictions about transmission efficiency, including the challenges presented by urban heat islands and climate change. However, the knowledge of temperature and chikungunya (CHIKV) dynamics within its two primary vectors—*Ae. aegypti* and *Ae. albopictus*—remains less characterized, even though CHIKV remains a virus of public-health importance. Here, we review the literature and summarize the state of the literature on CHIKV and temperature dependence of vector competence and EIP and use these data to demonstrate how the remaining knowledge gap might confound the ability to adequately predict and, thus, prepare for future outbreaks.

## 1. Introduction

Chikungunya virus (CHIKV) (family *Togaviridae*, genus *Alphavirus*) is a significant human pathogen that was originally isolated during an outbreak of dengue fever-like illness in Tanzania in 1952 [1]. While DENV was the primary cause of disease in this arbovirus outbreak, some afflicted individuals experienced arthralgia so severe that they were unable to straighten their bodies without assistance, leading to the adoption of the term “chikungunya”—meaning ‘to become contorted’ in the local Kimakonde language—as the official name of the virus [2]. Attempts at viral isolation using lab mice resulted in an unexpectedly high mortality for DENV, which suggested an alternative etiology and resulted in the isolation of CHIKV [3]. Historical review of past disease outbreaks suggests that CHIKV, misidentified as DENV, had been the causal agent of outbreaks in North America, India, Africa, and Asia since 1779 [4], primarily limited to tropical and sub-tropical regions with temperatures above the lower tolerance limit of the presumed vector(s), between 10 and 16 °C [5,6,7]. Since this definitive identification of CHIKV in 1950’s Tanzania, an identified CHIKV outbreak has occurred at least once every decade [8,9].

There are three lineages of CHIKV: the West African (WA) genotype, the Asian genotype, and the East/Central/South African (ECSA) genotype, which has a sub-lineage (the Indian Ocean Lineage, IOL) [9,10]. Aside from geographical range, it has been shown that there may be some difference in the levels asymptomatic presentation among the lineages, among the genomes—particularly in the untranslated regions and non-structural protein 3—and in the fitness of the IOL variant in *Aedes albopictus* [9,10].

As a zoonotic arbovirus, CHIKV is maintained in two transmission cycles: a sylvatic cycle involving (presumably) non-human primates and associated vectors, and an urban cycle involving humans and associated mosquitoes [11]. In both cycles, it is believed that *Ae.* spp. are the primary vectors, with primatophilic species acting as the major vectors in the sylvatic cycle, and the anthropophilic *Ae. aegypti* are considered the primary vector in most urban cycles; although *Ae. albopictus* can be either a secondary or primary vector, depending on multiple factors, including vector availability and environmental conditions [12]. In fact, in 2005, during an outbreak of ECSA CHIKV on La Réunion island, *Ae. Albopictus* was the primary vector due to a higher rate of contact because of a unique habitat niche on the island [13]. Although vector competence had previously been established, the high attack rate during this outbreak was outside of the predicted capacity of *Ae. Albopictus*-driven transmission. Subsequently, a mutation in the CHIKV genome was identified, which resulted in an amino acid change in the envelope protein (E1 A226V), and further examination of this mutation demonstrated an increase in CHIKV viral fitness in *Ae. albopictus*, with a higher vector competence for this strain compared to CHIKV strains without the mutation [14,15]. Interestingly, no change in viral fitness within *Ae. aegypti* was observed [15,16], suggesting that both vectors are competent for the virus, especially in tropical environments. Indeed, CHIKV was first reported in the Caribbean and South America in 2014 [17], and the following epidemic spanned 50 territories and generated nearly one million cases, with the Asian lineage as the predominant circulating lineage [9]. Low-level CHIKV transmission continues to be reported in South America and the Caribbean, indicating that it has become endemic in this tropical region [18].

However, with warming temperatures, the vector range of these two species has expanded and is predicted to continue to expand [19]. In 2007, another CHIKV ECSA A226V *Ae. Albopictus*-driven outbreak occurred, infecting 205 people in Italy [20], and there have been continued sporadic outbreaks in non-tropical areas including Italy, France, and more temperate regions of North and South America [21,22,23,24]. This encroachment out of the tropics has highlighted the importance of investigating CHIKV transmission across a wide range of temperatures, for both *Ae. aegypti* and *albopictus*, in order to understand potential patterns of transmission in known and new areas, as temperatures are expected to continue to deviate from past norms.

Research on the role of temperature on the within-vector dynamics of arboviruses has revealed that both vector competence and the extrinsic incubation period (EIP) are affected by both rearing and extrinsic incubation temperatures (EIT) [25,26,27,28,29,30,31,32,33]. Temperature has been linked to increases in the transmission efficiency of West Nile virus [34], Rift Valley Fever virus [35], Mayaro virus [36], dengue virus [37], and others [38]. In general, as temperature increases, vector competence increases and the EIP decreases [38]. However, the role of temperature is not monotonic, as one study demonstrated increases in temperature eventually led to decreases in vectorial capacity due to other factors such as mosquito mortality and biting [39,40,41]. Vector competence and EIP, however, remain foci of investigating the role of temperature for within-mosquito viral kinetics [27].

Vector competence and EIP have been used to explain transmission differences for CHIKV in *Ae. albopictus* when these measures were compared at discrete timepoints (i.e., significant differences found at 7 dpi) [15]. In addition, vector competence and EIP are impactful parameters of the vectorial capacity equation, which is a measure of transmission efficiency and a component of the basic reproductive number (R_0_) for vector-borne pathogens [13,15,42,43,44,45,46,47]. Thus, understanding the totality of vector competence, EIP, and the effects of temperature/climate change is critical for predicting (re-)emergence or expansion of this virus. This is particularly important as global warming predicts that more and more regions will be at risk for *Aedes*-driven arboviral transmission, including the southern continental United States [48]. However, the state of the literature regarding CHIKV and the effects of temperature has not been collated. Herein, we reviewed all available CHIKV vector competence–temperature data to assess the state-of-the-art of the literature and identify gaps in our knowledge.

## 2. Materials and Methods

A systematic search of CHIKV was conducted in PubMed through May 2023 to update data regarding vector competence and temperature. The search terms (“vector competence”, “extrinsic incubation period”, “temperature”, “chikungunya”) revealed a total of 90 hits. The inclusion criteria were defined as experimental papers on chikungunya in either *Aedes aegypti* and/or *Ae. albopictus* that included different EIT experimental treatment conditions and reported dissemination and/or transmission rates. Exclusion criteria were defined as duplicate papers, transgenic mosquitoes, modeling papers with no primary data and reviews, and papers where the temperature was not explicitly and numerically stated (e.g., “High DTR”). Data were extracted from the text, tables, or figures as available. Where applicable, data were extracted using PlotDigitizer (version 2.6.8). Data of interest included dissemination data (and what tissue was used for this determination), transmission data, mosquito species and source, chikungunya strain and genotype, and titer of exposure. Comparisons of the average transmission versus dissemination rates were performed using the Kolmogorov–Smirnov goodness-of-fit Test (when more than one timepoint was available to test the distribution of data) or via Mann–Whitney–Wilcoxon test (when only one time-point was available), and only in instances where both types of data were available (see Results). Data are shown in Supplemental Figure S1 and Supplemental Table S1.

In order to demonstrate the potential consequences of temperature-driven CHIKV dynamics, a compartmental Susceptible-Exposed-Infectious (SIE) model was constructed to simulate the introduction of one index case human. The EIP defines the rate of movement of mosquitoes from exposed to infectious, and we assumed the EIP follows an exponential distribution. Six temperature–species scenarios had enough data to fit a function in efforts to determine the EIP_50_. EIP_50_ is the time it takes for 50% of mosquitoes to reach infectiousness. Because dissemination rates were the most consistently reported metrics, we used these data to fit either a linear or exponential function to determine EIP_50_.

Of the six scenarios, three reached up-to or near EIP50, and simulations were run for these. Because not every scenario reached 50% within the timeframe of the studies, an additional approach was thus used, parameterizing the model with the EIP_MAX_ and associated dissemination rate for each scenario. The EIP_MAX_ is the time at which the highest rate of dissemination is observed and this was directly taken from the compiled data.

The system of equations is given below:

(1) $$\frac{dS_{H}}{dt}=S_{H}-a*S_{H}*\frac{I_{M}}{N_{H}}$$

(2) $$\frac{dE_{H}}{dt}=a*S_{H}*\frac{I_{M}}{N_{H}}-\sigma *E_{H}$$

(3) $$\frac{dI_{H}}{dt}=\sigma *E_{H}-\gamma I_{H}$$

(4) $$\frac{dR_{H}}{dt}=\gamma I_{H}$$

(5) $$\frac{dS_{M}}{dt}=E_{M}-\mu_{M}S_{M}-a*S_{M}*\frac{I_{H}}{N_{H}}*b$$

(6) $$\frac{dE_{M}}{dt}=a*S_{M}*\frac{I_{H}}{N_{H}}*b-\mu *E_{M}-EIP*E_{M}$$

(7) $$\frac{dI_{M}}{dt}=EIP*E_{M}-\mu *I_{M}$$

where a is the biting rate, μ is mosquito mortality, EIP is either EIP_MIN_ or EIP_50_ depending on the scenario, σ is the incubation period in the human, and γ is the human infectious period. Current model frameworks assume an exponential distribution to describe the movement of mosquitoes from exposed to infectious classes. However, this does not allow for granularity in vector viral kinetics [49,50]. Thus, an additional parameter, b, was used to represent the probability that a mosquito was infectious at the time of bite, as a proxy for vector competence. The transition rates are summarized in Supplemental Table S2.

Temperature-dependent mosquito mortality (μ) and bite rates (a) were varied according to [51] at the midpoint of the temperature classes (see Results), and all else was held constant. Since dissemination data were more consistently available, our model utilized these data for comparisons. The birth and death rates of the human population were not included, and a constant population size was assumed. The parameter values are given below in Table 1 and Table 2. The model was run for a total of 1000 simulations per scenario. For each scenario, the proportion of simulations that resulted in at least one secondary human transmission event was calculated and reported as the probability of autochthonous transmission. Next, the simulations with successful secondary transmission were temporally centered around the peak number of cases. The time-to-peak (in days) and peak number of cases were compared across the three scenarios. Stochastic realizations of the model were simulated using an algorithm that implements the tau-leap approximation to Gillespie’s algorithm with a time step of 0.125 [52]. All calculations and modeling were performed using R Studio (2022.07.0 Build 548) with R version 4.2.1.

## 3. Results

### 3.1. State of the Literature

**Figure 1:**
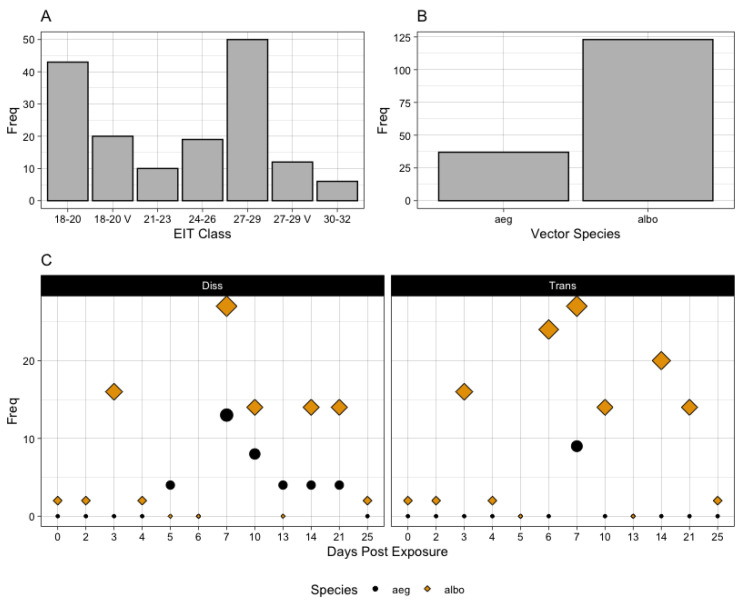
Out of the 8 publications examining the temperature dependence of CHIKV vector competence/EIP, (**A**) the frequency at which investigations were performed over each temperature category; (**B**) the frequency at which each mosquito species was investigated; (**C**) frequency of data at each recorded day post exposure according to vector species and the metric (dissemination or transmission).

After title and abstract review, thirteen papers were selected for full paper review [25,26,27,28,29,30,31,32,33,45,54,55]. Subsequently, a total of eight studies met the inclusion criteria of providing dissemination and/or transmission rates [25,26,27,28,29,30,32,33]. Of the eight studies, six looked at only constant temperatures [26,27,28,30,32,33] while two studies looked at both fluctuating and constant temperatures [25,29]. The distribution of temperature ranges considered is shown in Figure 1A. Only one study included both *Ae. aegypti* and *Ae. albopictus* [25]; two studies exclusively considered *aegypti* [26,28], while five studies exclusively studied *albopictus* [27,29,30,32,33] (Figure 1B). Of note, only one study investigated mosquitoes in the United States [25]. One paper investigated an Asian strain of CHIKV, one paper compared one Asian strain against an East-Central South African (ECSA) strain, and the others (n = 6) investigated ECSA strains only.

The distribution of timepoints investigated in all eight studies demonstrates a bias towards the 6–10 period, with another bump at 14 and 21 days (Figure 1C). Importantly, transmission data were available for *Ae. aegypti* at day 7 for only one study [25], while dissemination data were available across all studies and species. Transmission assays were performed in five studies [25,27,29,30,32]. In two studies, a subset of the temperature treatment information was generalized to a measure of variability (i.e., “daily temperature range”) without explicit ranges provided [25,29]. Six studies measured both dissemination and transmission rates [25,29,30,32,33] while two studies measured only dissemination [26,28], and one study only transmission [27]. Figure 1C shows the distribution of these metrics over time according to vector species. In 5/8 studies, the EIP was not explicitly discussed or interpreted [25,27,28,32,33], indicating that the interpretation of vector competence data in the context of its temporal process remains underrepresented in the literature.

### 3.2. State of Knowledge Regarding Temperature Dependence and In Vivo Dynamics in Aedes aegypti and albopictus

**Figure 2:**
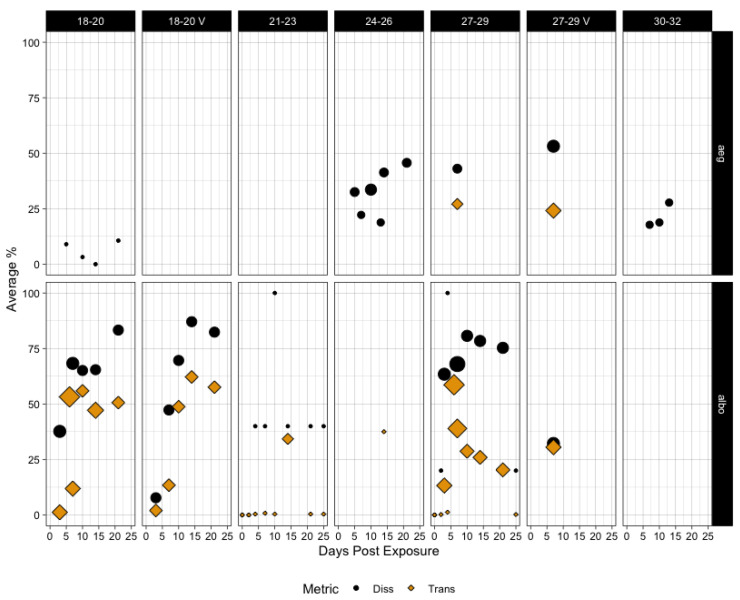
The average rate of dissemination (black circles) or transmission (gold diamonds) for each species across the combined temperature categories. Size of the points represents the number of data points at each time point/temperature across all 8 studies. V means variable (fluctuating) temperature with a mean in that temperature class.

Figure 2 shows the average dissemination and/or transmission for each temperature class per mosquito species per timepoint. Where available, dissemination and transmission were compared. Transmission rates were uniformly lower than dissemination rates when compared for *aegypti,* although not always significantly different: 27–29 °C (*p*-value = 0.7) and 27–29 °C-Variable (*p*-value = 0.002). The distribution of dissemination rates tended to be significantly higher in *albopictus*, as well: 18–20 °C (*p*-value = 0.01293), 18–20 °C-Variable (*p*-value = 0.035), 21–23° (*p*-value = 0.022), and 27–29 °C (*p*-value < 0.0001). At 27–29 °C-Variable, dissemination and transmission at day 7 were not significantly different (*p*-value = 0.94).

### 3.3. Functional Fits to Available Data

**Figure 3:**
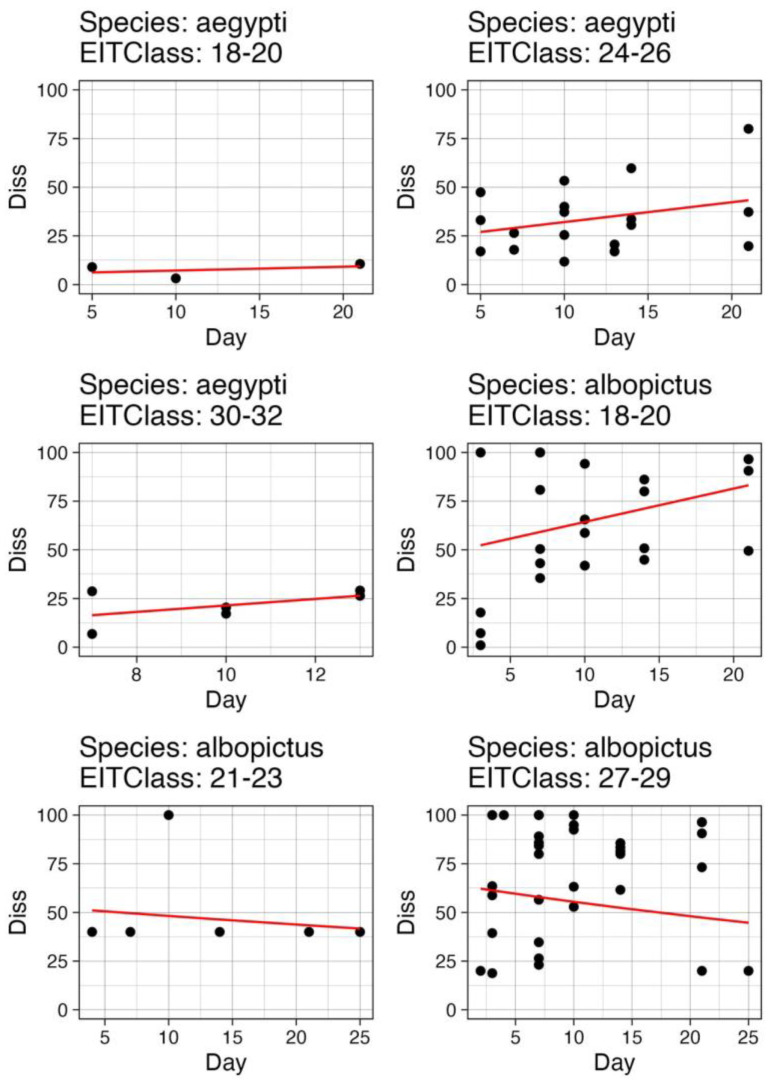
Functions fitted to available data of dissemination rates at each timepoint for each of the six scenarios considered (EITClass–species combinations). Y-axis is the percent dissemination and day is the day post exposure of mosquitoes from literature. See Table 3 for details about the functional forms and parameters for each scenario.

The six scenarios for which sufficient data were available to fit functions (Table 3) are shown in Figure 3. Using these functions, we were able to discern whether the EIP_50_ was reached within the timeframe of 25 days (timespan over which data were available and representing a reasonable mosquito lifespan). We found that in only three of the scenarios was EIP_50_ reasonably expected, and the resulting calculation of EIP_50_ is given in Table 4. The EIP_MAX_ was recorded directly from the data and is also given in Table 4 for all six scenarios.

### 3.4. Ae. aegypti-CHIKV Transmission and Temperature

**Figure 4:**
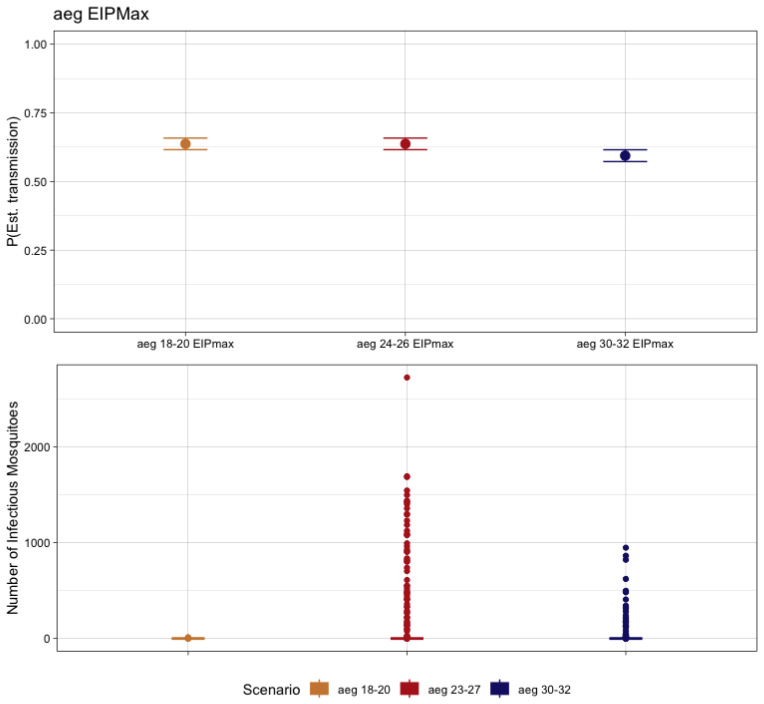
The probability of autochthonous transmission (**upper**) and number of infectious mosquitoes (**lower**) for CHIKV resulting from 1000 transmission simulations considering the EIP_MAX_ for the respective EITClass in *Ae. aegypti*.

Interestingly, the data available for *Ae aegypti*-CHIKV transmission systems did not indicate robust outbreak likelihoods. All three scenarios resulted in a relatively high probability of at least one human infection, with 18–20 °C having a probability of 63.7% of autochthonous transmission, 63.8% for 24–26 °C, and 59.5% for 30–32 °C. However, the scale of autochthonous transmission varied among temperature classes. Of the simulations that produced at least one locally acquired mosquito infection for the EITClass 18–20 °C, 97.96% (n = 624) produced no secondarily infected mosquitoes. The remaining 1.1% ranged from 1 to 13 secondarily infected mosquitoes. For the EITClass 24–26 °C, 79.9% (n = 510) produced no further transmission to a second mosquito generation and the remaining 20.1% (n = 128) ranged from 1 to 2722 secondarily infected mosquitoes. Finally, for the EITClass 30–32 °C, 83.2% (n = 495) of the simulations that resulted in at least one human case resulted in no secondarily infected mosquitoes. Of the remaining simulations, the range of infectious mosquitoes was from 1 to 947 (Figure 4).

### 3.5. Ae. albopictus-CHIKV Transmission and Temperature

**Figure 5:**
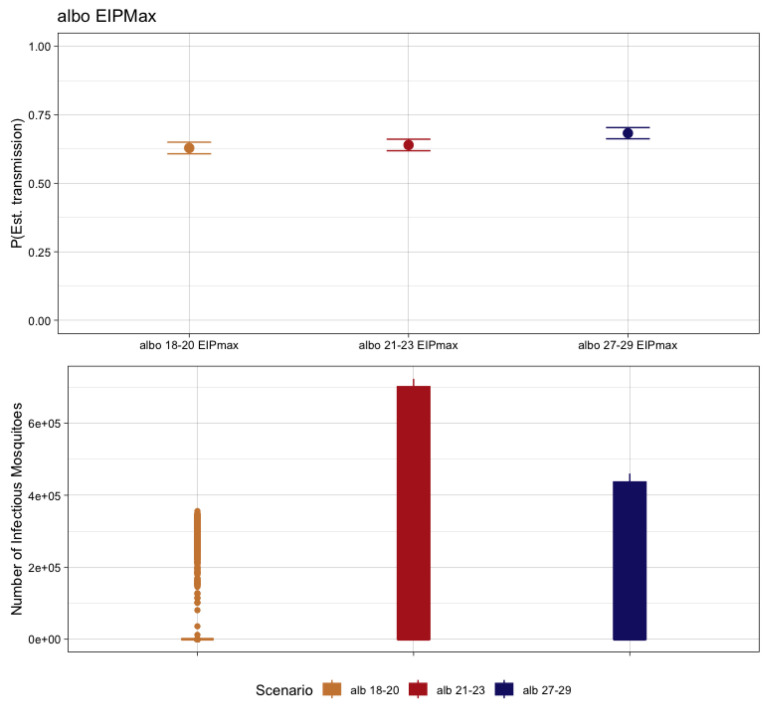
The probability of autochthonous transmission (**upper**) and number of infectious mosquitoes (**lower**) for CHIKV resulting from 1000 transmission simulations considering the EI_MAX_ for the respective EITClass in *Ae. albopictus*.

When we consider the EIP_MAX_ for comparing temperature-dependent transmission scenarios, the probability of at least one infected human was 63.4% for 18–20 °C, 64.0% for 22–24 °C, and 68.4% at 28–30 °C. Considering the scale of outbreak, again variability was observed. For the 18–20 °C EITClass, 65.0% (n = 412) of simulations with at least one human case produced no further transmission to mosquitoes. The range of mosquitoes for the remaining simulations that did produce forward transmission ranged from 1 to 356,143 over the year. For the 22–24 °C EITClass, 50.2% (n = 321) of those simulations with autochthonous transmission did not result in forward transmission to mosquitoes. The range of those that did produce infectious mosquitoes ranged from 1 to 723,473. Lastly, for the EITClass of 28–30 °C, only 36.4% of simulations with autochthonous transmission had no further transmission to mosquitoes (n = 249), and the remaining had a range of infectious mosquitoes of from 1 to 460,262 over the course of the year (Figure 5).

**Figure 6:**
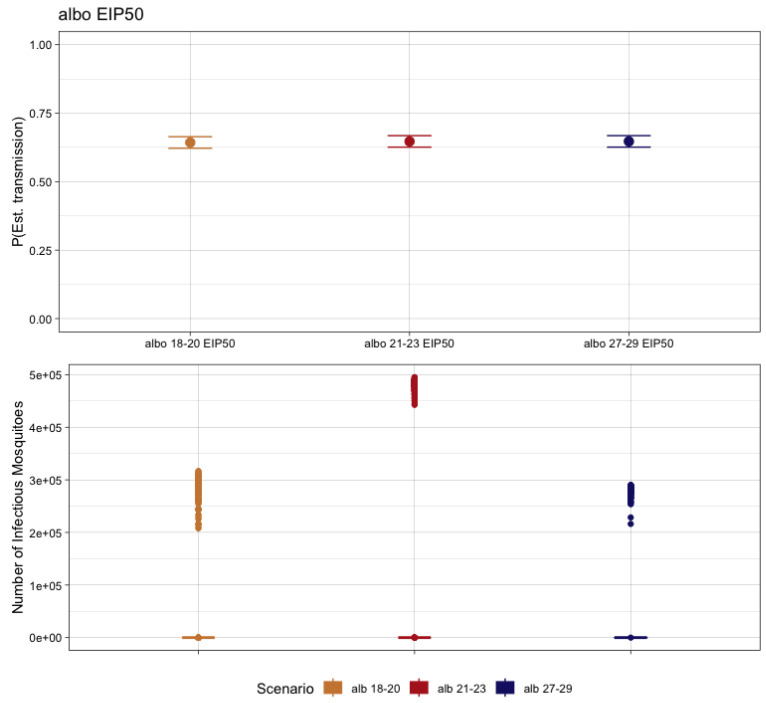
The probability of autochthonous transmission (**upper**) and number of infectious mosquitoes (**lower**) for CHIKV resulting from 1000 transmission simulations considering the EIP_50_ for the respective EITClass in *Ae. albopictus*.

When we considered EIP_50_ for *Ae. albopictus*, the probability of at least one human case was 64.3% for 18–20 °C, 64.7% for 22–24 °C, and 64.7% for 28–30 °C. For the 18–20 °C EITClass, 74.3% (n = 478) of simulations with at least one human case produced no further transmission to mosquitoes. The range of mosquitoes for the remaining simulations that did produce forward transmission ranged from 1 to 316,803 over the year. For the 22–24 °C EITClass, 65.8% (n = 426) of those simulations with autochthonous transmission did not result in forward transmission to mosquitoes. The range of those that did produce infectious mosquitoes ranged from 1 to 495,232. Lastly, for the EITClass of 28–30 °C, 61.2% of simulations with autochthonous transmission had no further transmission to mosquitoes (n = 396) and the remaining had a range of infectious mosquitoes of from 1 to 290,365 over the course of the year (Figure 6).

### 3.6. Temperature and Titer

**Figure 7:**
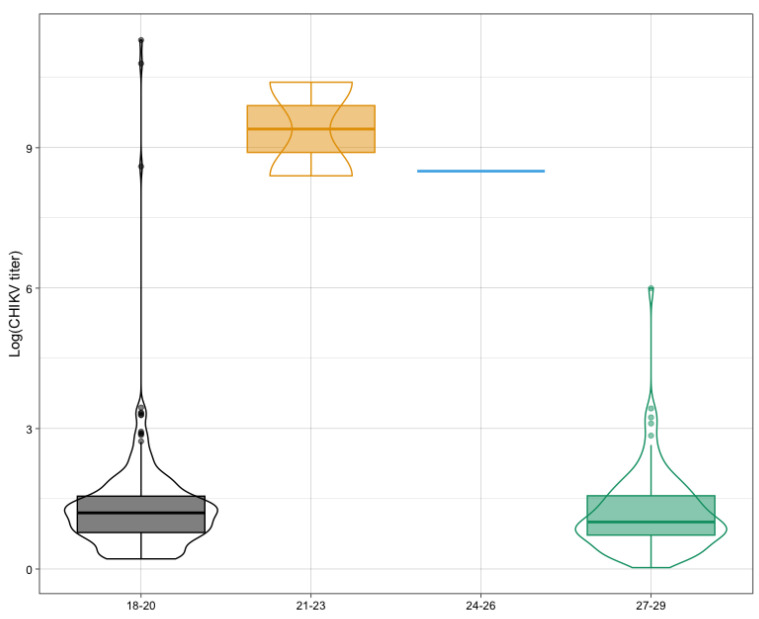
Summary of the available data regarding temperature dependence of CHIKV titer in *Ae. albopictus*.

Two studies have addressed the quantification of CHIKV in saliva with respect to temperature differences. Both studies were conducted in the vector *Ae. albopictus.* One study focused on this at 14 days post exposure [27] and one studied the effect of temperature on titer longitudinally [29]. Combining the data, we determined that there was not a significant difference in time points (*p* = 0.237, analysis of variance), but there was a significant effect of temperature (*p* < 0.0001). The interaction term was not significant (*p* = 0.460), indicating that the main effect of temperature drives differences. Figure 7 shows the data from these two studies. While time was not significant, replication of these studies is needed to verify this null effect and parse out the extended role of temperature on CHIKV titer and the effects on transmission. Notably, no studies have undertaken the study of this phenomenon in *Ae. aegypti*.

## 4. Discussion

The surprisingly low volume of published literature related to this topic demonstrates the under-representation of the knowledge around the CHIV and temperature relationship. One possible reason for this is the biosafety resources necessary to study CHIKV safely. CHIKV is classified as a Risk Group 3 organism and it requires a Biosafety Level-3 (BSL-3) and associated arthropod containment level-3 (ACL-3) laboratories for safe handling in many countries [56]. While such enhanced biosafety is important and should be utilized, it does mean that a limited number of laboratories are available within which to work, and that expertise is even more limited at the interface of high containment and arbovirus research.

However, another potential reason for this phenomenon is the large (almost overwhelming) body of knowledge about the relationship between temperature and *Aedes* spp. and, in addition, how it affects transmission of other arboviruses, especially those that require more accessible BSL-2 laboratories. Most of these works focus on DENV and, hence, a widely generalized presumption is that these trends and correlations are extrapolatable for other arboviruses. Nonetheless, it has been shown that interactions between strains of the same virus genotypes shape traits like vector competence [33,57]. This suggests that by extrapolating from a totally different virus, the real variability of the temperature dependence of CHIKV-vector kinetics and the impact of such remains clouded. This is particularly true for *Ae. aegypti*, which is the species likely responsible for most transmission in tropical regions [58,59], as the surprisingly little published data are not representative of what could be happening in nature. Despite this, the fact that our results show a contribution of *Ae. aegypti* in transmission, even if it is much lower than *Ae. albopictus*, highlights the importance of transmission, even for additional transmission-related phenomena like of viral circulation continuing in nature.

For example, the gulf states of the U.S. are at-risk for such outbreaks, especially as climate change also drives the increase in natural disasters such as hurricanes and floods. In the last 50 years, the average temperature in New Orleans has risen 0.8 °F and Lafayette has risen 0.5 °F [60]. A climate report recently indicated that Lafayette and Baton Rouge were estimated to have had an increase of 2.5% in the number of days suitable for mosquito activity given rising temperatures from 1970 to 2017. New Orleans and Lake Charles were predicted to have had an 3.8% and 3.0% increase, respectively [22,60]. This risk is in terms of temperature only and does not add to the exacerbation of natural disasters. In the last 10 years alone, South Louisiana has experienced five major hurricanes, one tropical storm, and a major flooding event that caused significant stress on infrastructure and an interruption of vector control in the affected areas [21]. For example, following Hurricane Irma landfall in Florida, an increase in mosquito–human encounters were proposed to be in part from “increased outdoor activity during cleanup efforts, open windows due to the lack of air conditioning, and/or a lack of familiarity with…mosquito densities by out-of-state contractors [4]”. Following a large flooding event in South Texas in 2018, mosquito “hot spots” were identified in areas of ongoing recovery efforts [61]. Hurricanes in Louisiana have an impact on existing mosquito-borne disease systems. There was an increase in neuroinvasive West Nile disease incidence of 94.3% across areas with hurricane-induced damage [62]. Mosquitoes and the risk they pose to public health as disease vectors was highlighted in 2018 [63] by the U.S. Centers for Disease Control and Prevention and the American Mosquito Control Association in an effort to devise the first-ever guidance for mosquito control after natural disasters [64]. In this management plan, it is mentioned that the continued collection of arbovirus data is one of the three main activities that comprise “mosquito management emergency response [63]”. While these recommendations are tailored for surveillance in an operational setting, understanding the baseline of CHIKV risk in non-emergency settings, and its potential to increase with warming temperatures, further facilitates preparedness and rapid response.

And yet, *Aedes*-borne diseases have not become endemic in most of the Southern United States, despite travel-associated cases, occasional autochthonous transmission, and competent mosquitoes [48,65,66,67,68,69,70,71,72]. This has been attributed mainly to the lifestyle of air conditioning, screens, and other mosquito-avoidance infrastructure [73]. However, there is a growing health disparity in the South as homelessness and suboptimal housing is increasing [74,75]. In 2020, there were approximately 3200 homeless individuals in Louisiana with 1314 (41.4%) of them in the greater New Orleans area alone [76]. According to a United States congressional report on homelessness in 2022, that number had grown to 7373, and in 2023 there were 1390 homeless people in New Orleans (an increase of 5.8%) [77]. With homeless encampments on the rise, this brings the added factor of trash piles, which are known breeding source for *Aedes* mosquitoes [78,79]. In addition to homelessness, substandard housing in areas of poverty often means a lack of mosquito-contact interruption such as air conditioning as well as increased trash dumping [79], making impoverished and homeless individuals more at-risk in the Southern U.S. Despite robust vector-control programs, during the Zika epidemic, it was recognized that the over 8 million individuals living in poverty along the Gulf Coast were at enhanced risk for *Aedes*-borne diseases (Hotez in [80]). Thus, there is a need not only for prophylactic research into the emergence potential of *Aedes-*borne viruses, such as chikungunya for disaster planning, but also to address growing health disparities.

Arbovirus transmission in Kenya is another example of the importance of understanding CHIKV transmission and the thermal pressures that may alter trajectories. Mosquitoes from the region were shown to be competent for CHIKV [81], and more recent reports have shown that CHIKV was almost 15 times more prevalent in *Ae. aegypti* collected in Kenyan cities compared to DENV [82]. Further, serosurveys have demonstrated higher seroprevalence for CHIKV compared to DENV in the human population [83,84,85]. What is currently expected of arbovirus transmission will change as climate changes and temperatures vary from what has been the norm, and urbanization (which also drives temperature increases at multiple scales) will continue to play a role in transmission, as well as other social determinants of health that are likewise affected by climate change, including food security, housing security, etcetera [86,87,88]. Thus, for places where CHIKV has already emerged—like Kenya, for places where the burden will be altered—such as in Southern Europe and Sub-Saharan Africa, and for places it has yet to emerge—such as the Gulf South, understanding transmission is paramount. However, this large gap in a known affecter of transmission—temperature—remains understudied. Therefore, the ability to prevent, prepare for, and respond to outbreaks is hindered.

The data herein likely do not represent the prevailing phenotype(s) nor the diversity of phenotypes for CHIKV for these temperature ranges, as our state of the data indicates how little is published regarding this interaction among the virus, the vector, and the external factor of temperature. What the model outputs from the available data do show is that even with the paucity of data, it is possible to demonstrate that there is likely to be diversity in how an outbreak behaves based on temperature effects. Critically, more data are needed for the community to understand the breadth of possibilities of CHIKV transmission trajectories, especially given that vector ranges are expanding, and temperatures are variable across the globe [89,90].

There is value to more comprehensive understanding of virus–vector interactions, especially as they relate to external pressures from climate change and, especially, temperature. With only eight papers reporting temperature-driven dynamics, our review demonstrates that there is a gaping hole in our knowledge of chikungunya and the role that temperature and climate change may play in its (re)emergence and expansion. As temperature continues to play a major role in shaping the transmission of arboviruses, climate change will drive vector range expansion [19,91], resulting in risk for both the population at large and more vulnerable groups across the globe.

## Figures and Tables

**Table 1:** Temperature-dependent longevity and biting rate of adult *Ae. aegypti* and *albopictus* from [51] (Mordecai et al. 2017) .

Species	Temperature	Longevity (days)	Biting Rate (1/day)
*Ae. aegypti*	19 °C	26.6	0.09
	22 °C	29.2	0.15
	25 °C	29.3	0.22
	28 °C	26.5	0.28
	31 °C	21.1	0.32
*Ae. albopictus*	19 °C	95.0	0.14
	22 °C	110.5	0.20
	25 °C	102.5	0.25
	28 °C	68.9	0.30
	31 °C	13.4	0.33

**Table 2:** Constant parameters used in the model.

Parameter	Value
Mosquito Emergence Rate	5000/7 days
P (Transmission|Bite) from mosquito to human	1
Human Incubation Period	5 days [53]
Human Infectious Period	8.5 days [53]

**Table 3:** Model functional forms and parameters to determine whether each scenario reached EIP50 in a reasonable amount of time considering mosquito lifespan. In only three scenarios was EIP50 reached, and these are marked with a *.

Species	EITClass	Fit	Parameters
*Ae. aegypti*	18–20	Linear	Int = 5.269 Slope = 0.195
	24–26	Linear	Int = 21.910 Slope = 1.019
	30–32	Linear	Int = 4.767 Slope = 1.667
*Ae. albopictus*	18–20 *	Linear	Int = 47.185 Slope = 1.713
	21–23 *	Exponential	Start = 53.059 Rate = −0.0096
	27–29 *	Exponential	Start = 64.044 Rate = −0.014

**Table 4:** The EIP_50_ (where appropriate) where 50% of mosquitoes are expected to transmit, and the EIP_MAX_ and associated dissemination proportions (vector competence proxy) for the mosquito species—EIT Class scenarios.

Mosquito Species	EIT Class	EIP_MAX_ (Dissemination %)	EIP_50_
*Ae. aegypti*	18–20	21 (10.6%)	NA
	24–26	21 (45.7%)	NA
	30–32	13 (27.7%)	NA
*Ae. albopictus*	18–20	21 (83.3%)	1.643
	21–23	10 (100%)	6.176
	27–29	4 (100%)	17.213

## Data Availability

All data is included in Supplemental Information and was generated from published literature. Other materials are available upon reasonable request to the corresponding author.

## Supplementary Materials

The following supporting information can be downloaded at: https://www.mdpi.com/article/10.3390/pathogens12111368/s1, Figure S1: Data Summary; Table S1: Data Summary; Table S2: Model Transition Rates Summary.
