# Supplementary material for: Identifying Knowledge Gaps through the Systematic Review of Temperature-Driven Variability in the Competence of Aedes aegypti and Ae. albopictus for Chikungunya Virus
Source: Pathogens. 2023 Nov 18;12(11):1368. doi: 10.3390/pathogens12111368 (PMC10675276; doi:10.3390/pathogens12111368)
Supplement: Supplementary file 1 [file pathogens-12-01368-s001.zip › pathogens-2721246-supplementary.pdf]

Supplemental Information: Identifying knowledge gaps through the systematic review of temperature-driven variability in the competence of *Aedes aegypti* and *Ae. albopictus* for chikungunya virus

RC Christofferson et al.

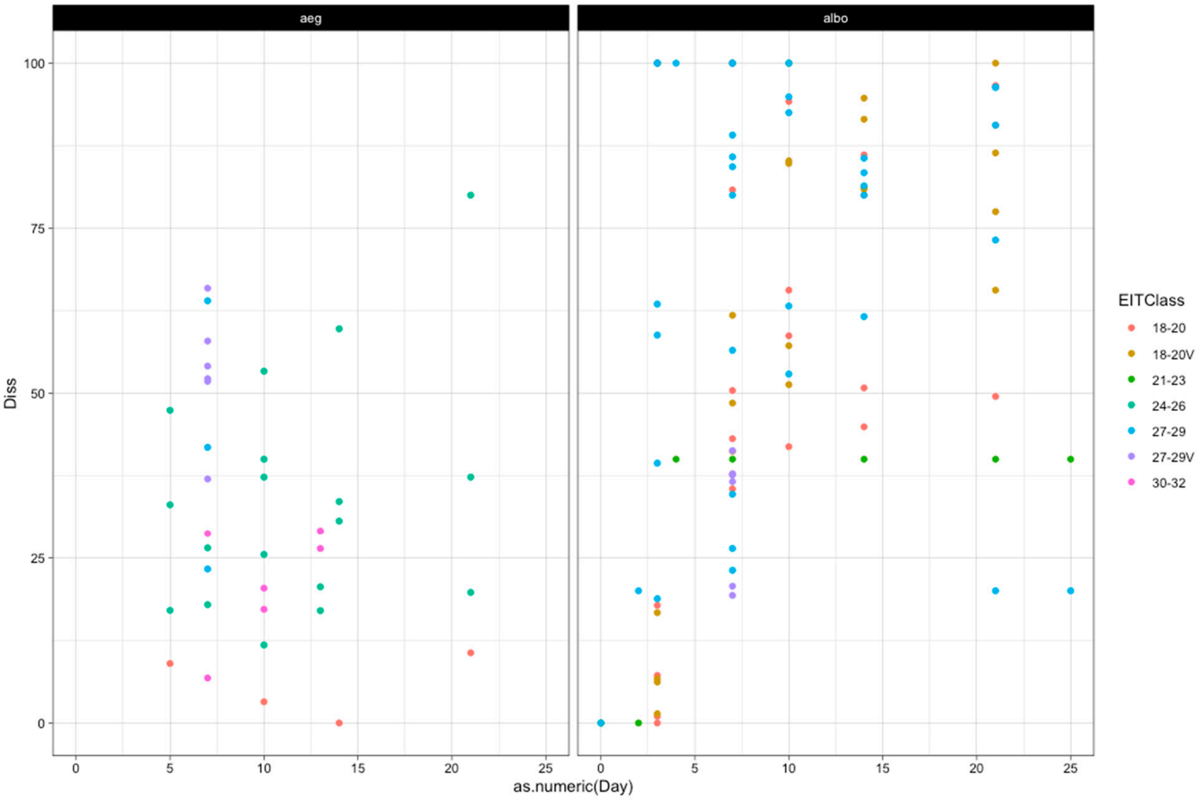

Supplemental Figure S1: Data Summary: A scatterplot of all data points collated from the papers included in this systematic review.

Supplemental Table S1: Data Summary. Summary of the day-by-day dissemination of CHIKV with respect to EITClass and species.

EITClass	Species	Day Post Exposure	Mean Dissemination	Number of data points
18-20	aeg	5	9	1
18-20	aeg	10	3.209877	1
18-20	aeg	21	10.617284	1
18-20	albo	3	45.2	5
18-20	albo	7	68.3	6
18-20	albo	10	65.1	4
18-20	albo	14	65.45	4
18-20	albo	21	83.3	4
18-20 V	albo	3	7.75	4
18-20 V	albo	7	47.3	4
18-20 V	albo	10	69.625	4
18-20 V	albo	14	87.075	4
18-20 V	albo	21	82.375	4
21-23	albo	4	40	1
21-23	albo	7	40	1
21-23	albo	10	100	1
21-23	albo	14	40	1
21-23	albo	21	40	1
21-23	albo	25	40	1
24-26	aeg	5	32.510288	3
24-26	aeg	7	22.2	2
24-26	aeg	10	33.583456	5
24-26	aeg	13	18.8	2
24-26	aeg	14	41.316872	3
24-26	aeg	21	45.679012	3
27-29	aeg	7	43.033333	3
27-29	albo	2	20	1
27-29	albo	3	63.416667	6
27-29	albo	4	100	1
27-29	albo	7	67.99	10
27-29	albo	10	80.7	5
27-29	albo	14	78.4	5
27-29	albo	21	75.3	5
27-29	albo	25	20	1

27-29 V	aeg	7	53.15	6
27-29 V	albo	7	32.216667	6
30-32	aeg	7	17.75	2
30-32	aeg	10	18.8	2
30-32	aeg	13	27.75	2

Table S2: Model Transition Rates Summary: Events and corresponding transition rates in the stochastic SEIR-SEI model. For each event, we list only those states that change. Parameter values are given in the main text and in Tables 2-3 .

Event	Change in state	Transition rate
**Transmission from mosquito to human**	(Sh,Eh) → (Sh-1, Eh + 1)	$a * S_H * \frac{I_M}{N_H}$
**Onset of infectiousness in human**	(Eh,ID1) → (Eh-1, ID + 1)	$\sigma * E_H$
**Recovery in human**	(ID10,Rh) → (ID10-1, Rh + 1)	γI_H
**Adult (female) mosquito recruitment**	(Sm) → (Sm + 1)	E_M
**Susceptible mosquito death**	(Sm) → (Sm-1)	$\mu_M S_M$
**Transmission from human to mosquito**	(Sm,Em) → (Sm-1, Em + 1)	$a * S_M * \frac{I_H}{N_H} * b$
**Exposed mosquito death**	(Em) → (Em-1)	$\mu * E_M$
**Onset of infectiousness in mosquito**	(Em,Im) → (Em-1, Im + 1)	$EIP * E_M$
**Infectious mosquito death**	(Im) → (Im-1)	$\mu * I_M$
